# HMG-CoA reductase inhibitors in kidney transplant recipients receiving tacrolimus: statins not associated with improved patient or graft survival

**DOI:** 10.1186/1471-2369-11-5

**Published:** 2010-04-01

**Authors:** Nizar Younas, Christine M Wu, Ron Shapiro, Jerry McCauley, James Johnston, Henkie Tan, Amit Basu, Heidi Schaefer, Cynthia Smetanka, Wolfgang C Winkelmayer, Mark Unruh

**Affiliations:** 1 Department of Medicine, Renal-Electrolyte Division, University of Pittsburgh, Pittsburgh, PA, USA; 2 Department of Surgery, Starzl Transplantation Institute, University of Pittsburgh, Pittsburgh, PA, USA; 3 Division of Nephrology and Hypertension, Vanderbilt University, Nashville, Tennessee, USA; 4 Division of Nephrology, Stanford University School of Medicine Palo Alto, CA USA

## Abstract

**Background:**

The beneficial effects of early statin use in kidney transplant recipients, especially those on tacrolimus-based immunosuppression, are not well established. We evaluated the predictors of statin use following kidney transplantation and examined its association with patient and allograft survival.

**Methods:**

We examined 615 consecutive patients who underwent kidney transplant at our institution between January 1998 and January 2002. Statin use was assessed at baseline and 3, 6, 9, and 12 months following kidney transplant. Patients were followed for allograft and patient survival.

**Results:**

36% of the 615 kidney transplant recipients were treated with statin treatment. Statin use increased over the course of the study period. Older age, elevated body mass index, higher triglyceride levels, hypercholesterolemia, diabetes, history of myocardial infarction were associated with higher rates of statin use; elevated alkaline phosphatase levels and CMV IgG seropositivity were associated with less statin use. Older age, elevated BMI and hypercholesterolemia remained significant predictors of increased statin use after accounting for covariates using multiple regression. The early use of statins was not associated with improvements in unadjusted patient survival [HR 0.99; 95%CI 0.72-1.37] or graft survival [HR 0.97; 95% CI 0.76-1.24]. The risks of death and graft survival were not consistently reduced with exposure to statin using either adjusted models or propensity scores in Cox Proportional Hazards models.

**Conclusions:**

In a kidney transplant population primarily receiving tacrolimus-based immunosuppression, early statin use was not associated with significantly improved graft or patient survival.

## Background

Statins have known effects in reducing cardiac as well as overall mortality in the general population[1]. The benefits of statins in reducing overall mortality are not well established in patients with kidney disease, including the kidney transplant population. A randomized controlled trial in diabetic hemodialysis patients showed no improvement in survival in those patients randomized to receive atorvastatin compared to placebo[2]. The Assessment of LEscol Renal Transplantation (ALERT) study is the only randomized controlled trial of statins in kidney transplant patients[3]. This trial found no statistically significant benefit of fluvastatin compared to placebo in achieving the primary outcome of a reduction in major adverse cardiac events (MACE); in addition, no statistical difference was seen in the overall mortality or graft survival between the fluvastatin and placebo groups. However, a 2 year extension of the ALERT trial showed that patients randomized to the fluvastatin group had a reduced risk of MACE; there remained no significant difference in overall mortality and graft loss between the groups[4]. The findings of the ALERT trial suggest that the timing of statin use may determine the magnitude of the non-immune benefits of statins. There was a greater reduction in major cardiac events in those patients initiating statin therapy early (years 0-2) than in those initiating statin therapy late (>6 years) following transplantation[5]. A recently published observational study used the Austrian transplantation registry to analyze the efficacy of statins in kidney transplant patients[6]. This study showed improved patient survival but no improvement in graft survival in patients receiving statins.

While the cholesterol lowering effect of 3-Hydroxy-3-methyl glutaryl coenzyme A (HMG CoA) reductase inhibitors (statins) is well-established, recent reports suggest that there are an increasing number of extra-lipid effects, including various anti-inflammatory and immunomodulatory actions[7-9]. Possible mechanisms that have been proposed by which statins may specifically benefit the transplant population include the alteration of free drug levels, the modulation of thrombotic processes and endothelial function, and the effect on inflammatory and immune responses--both dependent and independent of inhibition of the HMG-CoA reductase enzyme[7,8,10,11]. Observational and experimental evidence in lung,[12] pancreas,[13] and heart [14-16] transplantation suggests that treatment with statins leads to an increased survival and fewer episodes of transplant rejection, although the mechanism by which this occurs has not been fully delineated. Despite these positive findings in extra-renal solid organ transplants, an immunologic benefit has not been clearly demonstrated following renal transplantation[3-5,17-25]. Early pilot and single-center retrospective studies found a positive effect of various statins on the incidence of acute rejection and patient survival in kidney transplantation[18,24].

There are many potential factors that may affect statin use in the clinical setting. We are not aware of any studies in the published literature examining predictors of statin use in kidney transplantation. Furthermore, in contrast to cyclosporine, tacrolimus is less likely to induce dyslipidemia, and thus lead to the initiation of statin therapy for lipid management[26]. Hence, the determinants of statin use in the kidney transplant population, and the extent to which there is a benefit of early statin use following kidney transplantation in the contemporary kidney transplantation population, particularly in those receiving tacrolimus based immunosuppression, remains unclear following the ALERT Study. In order to address these gaps in knowledge regarding the determinants and outcomes of statin use in kidney transplantation, this report expands the existing evidence by examining the effect of statins in an incident cohort of kidney transplant recipients managed predominantly with tacrolimus-based immunosuppression and assesses the extent to which early statin use is associated with benefit. First, this report evaluates factors associated with statin use in a community-based setting with contemporary immunosuppression. Second, we examine the potential benefits of using statins following kidney transplantation in this cohort of kidney transplant patients followed prospectively for medication use, and graft and patient survival.

## Methods

### Patient Population

In this historical cohort study, we examined all 715 consecutive patients who received a renal transplant from either deceased or living donors at our institution between January 1998 and January 2002 and include follow-up until July 2005. 615 patients who survived and had a functioning graft a year after transplantation were included in the analysis to avoid immortality time bias. Greater than ninety percent of patients received a tacrolimus based immunosuppressive regimen. An induction, initially with thymoglobulin, and later with alemtuzumab, and tacrolimus monotherapy protocol was introduced in July 2001, and 15% of the patients included in this study were treated with this regimen, as previously described[27,28]. Under this new protocol, additional agents, such as antimetabolites, are reserved for the management of patients experiencing episodes of rejection or deemed to be at high risk of rejection, at the discretion of the treating transplant physician. Sirolimus was used in 8.5% and azathioprine in 1% of our patients. Sixty-eight percent of patients received maintenance steroid therapy. The use of maintenance steroids decreased over the study period. Only 2 out of the 615 patients received cyclosporine based immunosuppression. Immunosuppresive medications were routinely evaluated by frequent blood level determinations and adjusted for clinical events without consideration of statin use.

### Human Subjects Protection

Information used for our analyses was obtained through an honest broker system from prospectively recorded databases maintained by the University of Pittsburgh Medical Center and Starzl Transplantation Institute, under the auspices of, and with formal approval by, the Institutional Review Board of the University of Pittsburgh (Pittsburgh, PA). Research data were coded to prevent the identification of subjects.

### Clinical Treatment

Choice and dosing of statins were prescribed at the discretion of both the physicians in our transplant clinic as well as the patients' non-transplant physicians. Lipid measures were obtained pre-operatively on the day of transplant and subsequently at the follow-up points as part of routine clinic blood work. At the time of this study, routine clinic blood work included only total cholesterol and triglyceride levels. Full lipid profiles were not available for the majority of our patients. Statin use was assessed in clinic as part of the routine detailed review with patients of their medications, conducted by a post-operative transplant coordinator at baseline and 3, 6, 9, and 12 months following transplantation. We classified patients with a significant exposure to statin therapy from those who discontinued the drug after only a brief exposure. There were 19 patients who received statins during only a single time point. For the purpose of this study, these patients were included with the control group. All patients recorded as receiving any statin at 2 or more time points, up to one year following transplantation, were assigned to the treatment group. Detailed information regarding pre-transplant statin use was not available.

### Data Collection

The primary outcome measures were graft failure and patient death. Graft failure was defined as loss of renal function requiring re-transplantation or initiation of long-term dialysis. Data were recorded into the transplant patient database by patient care coordinators. For patients whose grafts failed and who returned to dialysis, the coordinators relied on various sources including reports from outside nephrologists, dialysis units, patient families, and newspaper obituaries to update the vital status.

Baseline patient comorbidity was assessed using specific individual comorbid conditions as well as by using an adjusted Charlson comorbidity index to determine the effects of composite comorbidity on the specified outcomes, as previously described[29]. Additional covariates included patient demographics (age, gender, race), clinical factors (cause of ESRD, previous transplant, body mass index [BMI], BMI^2^, immunosuppressive protocol), and laboratory values (liver function tests, CMV and hepatitis B and C status, total cholesterol), year of transplantation, as well as donor factors (living or deceased, age, antigen matching) and transplant procedure characteristics (cold ischemia time and delayed graft function, defined as the need for dialysis in the first week following transplantation).

### Statistical analysis

Baseline demographic, laboratory, and transplant factors are described as means and standard deviations for continuous variables and as frequency distributions for dichotomous variables. Statistical significance of the differences between groups was tested using two-sample t-tests or ANOVA for continuous variables and Chi-square tests for categorical variables.

### Determinants of statin use

We created a multiple logistic regression model to identify factors independently associated with the use of statins following kidney transplantation. All covariates with p < 0.2 in the univariate analysis were entered initially, and the parameter estimates of predictors of statin exposure were examined after the removal of each potential covariate with p > 0.20 to assess for possible confounding. In the final model, race and gender were forced into the model, which included age, body mass index, liver disease, CMV IgG seropositivity, diabetes, MI, cholesterol, triglycerides, and history of previous transplant. In our analysis of predictors of statin use, a number of study patients lacked data for one or more variables. In order to assess the influence of missing data, we examined the multiple logistic regression parameter estimates using a complete case analysis, interpolating missing values, and substituting average values. Statin use increased over the study period; in order to adjust for this, we included both the year of transplant and a sequential transplant identification key into the regression model to assess whether it affected our analysis

### Survival analysis

We hypothesized that the use of statins would be associated with longer patient and graft survival. While statin use was assessed over the first year after transplantation, all analyses of graft and patient survival were conducted from one year after transplantation in order to avoid an immortality time bias[30]. Similarly, to decrease the effect of confounding by factors such as significant post-operative morbidity or serious co-morbid illness on the use of statins, we limited our survival analyses to patients who survived, and to grafts that functioned, at least one year. Patients lost to follow up and those remaining enrolled at the close of the study were censored at the time of those events. Univariate and multivariate Cox proportional hazards models were used for time-to-event analyses to assess the crude and independent associations between statin use and study outcomes. In order to adjust for baseline factors with potential to confound the relationship of statin use to subsequent outcomes, baseline covariates significantly associated with statin use and other major demographic covariates thought to be associated with survival were entered into the Cox proportional hazards regression model. We examined survival models using additional groups of variables to identify potential confounding. To account for the effect of baseline creatinine on graft survival, we adjusted for creatinine at 12 months when examining the association between statin use and graft survival. The components of our fully adjusted models varied by outcome but included statin use and those factors significantly associated with patient and graft survival: age, sex, race, and if appropriate, body mass index (BMI), BMI^2^, donor type, diabetes, history of previous transplant, CMV IgG seropositivity, tolerance protocol, creatinine at 12 months, history of myocardial infarction (MI), severe liver disease (SLD), and presence of delayed graft function (DGF). Propensity scores were generated via logistic regression modeling. All variables that were assumed to be associated with statins (treatment) were included. The hazard ratios for statin use versus no statin use were explored via the following methods and compared. (1) The inverse of the propensity scores were used as weights in a Cox proportional hazard model. (2) The propensity scores were included in a Cox proportional hazards model as a continuous covariate. (3) The propensity scores were ranked into quintiles and used as a covariate in a Cox proportional hazards model. The survivorship curves were then compared using log rank tests.

We performed a power calculation using our event rates for patient deaths and graft failures. Our study had eighty percent power to detect a 35% difference in patient survival between the statin and non-statin groups. The study also had at least 80% power to detect a 35% difference in graft failure rate between the two groups. All analyses were conducted using SAS software version 8.2 (SAS Institute Inc, Cary, NC). Data are presented as means ± SD and reported as significant if p < 0.05.

## Results

### Demographics

Baseline patient and donor characteristics are detailed in Table 1. Two hundred and twenty one (36%) of the 615 kidney transplant recipients met our definition of statin treatment. Mean ages in the statin-treated and non-statin-treated groups were 51 (+/- 12) and 48 (+/- 15) years, respectively (p = 0.002), and the majority of patients were male (62.4% and 63.3%, p = 0.82) and white (86.9% and 81.5%, p = 0.08; Table 1). Patients with older age, higher BMI, hypertriglyceridemia, hypercholesterolemia, diabetes complicated by end organ damage and history of myocardial infarction had higher rates of statin use. Patients who were CMV IGG positive and those who had higher alkaline phosphatase levels had lower rates of statin use. Among those with a complete lipid panel at 12 months (n = 72), there was no significant difference in LDL (statin and non-statin groups; 112(46) vs.97(34); p = 0.12) or HDL (statin and non-statin groups; 45.1(13.9) vs.45.4(16.3); p = 0.93). There was a trend towards greater statin use over the course of the study period. The use of statins was associated with year of kidney transplant, 22.9% (1998), 27.9% (1999), 47.1% (2000), 45.0% (2001), and 37.7% (2002), respectively (p < 0.001).

**Table 1:** Baseline patient, donor, and transplant procedure characteristics.

Characteristics	Statin (N = 221)	Non-statin (N = 394)	All (N = 615)	p-value
**Baseline patient characteristics**				
Age at transplant (y; N = 615) *	51 ± 12	48 ± 15	50	0.002
Male (%; N = 614)	62.4	63.3	63.0	0.82
Race (N = 615)				
White (%)	86.9	81.5	83.4	0.08
Other (%)	13.1	18.5	16.6	
Body Mass Index (kg/m^2^; N = 599) *	26 ± 5	25 + 5	26	0.002
Etiology of ESRD (%, N = 615)				
DM	18.6	13.4	15.3	0.12
HTN	21.7	17.3	18.9	
Other	50.7	59.1	56.1	
Unknown	9.0	10.1	9.8	
Charlson Co-morbidity Index				
Total score (N = 611)	2.13 ± 1.81	2.04 ± 1.90	2.08	0.60
Myocardial Infarction (%, N = 611)	9.9	5.6	7.2	0.05
Severe liver disease (%, N = 611) *	1.8	3.3	2.8	0.27
Previous Transplant (%, N = 615)	17.2	23.3	21.1	0.07
PRA (%; N = 590)	8.1 ± 18.8	9.0 ± 20.0	8.7	0.62
Liver function				
AST (U/L; N = 541)	22 ± 11	22 ± 13	22 ± 12	0.95
ALT (U/L; N = 542)	28 ± 19	28 ± 19	28 ± 19	0.95
Alkaline phosphatase (U/L; N = 540)*	97 ± 50	118 ± 113	110 ± 94	0.02
Tbili (mg/dL; N = 539)	0.5 ± 0.2	0.6 ± 0.4	0.6 ± 0.4	0.61
HbsAg positive (%; N = 615)	2.3	0.5	1.1	0.05
Anti-HCV positive (%; N = 615)	6.3	8.4	7.6	0.59
CMV IgG positive (%; N = 612) *	73.3	81.3	78.4	0.02
Total cholesterol (3 m) (mg/dL; N = 467) Triglycerides (mg/dL; N = 456)	200 ± 47 220 ± 124	181 ± 42 181 ± 118	188 197	< 0.001 < 0.001
Creatinine at 3 mos (mg/dL; N = 590)*	1.8 ± 0.7	1.8 ± 1.1	1.8	0.28
Diabetes with complications (N = 611)	21.3	14.4	16.9	0.03

**Donor characteristics**	**Statin (N = 163)**	**Non-statin (N = 552)**	**All (N = 715)**	**p-value**

Deceased donor (%; N = 615)*	73.3	75.9	75.0	0.47
Age (y; N = 594)	37 ± 17	37 ± 17	37 ± 17	0.88
A Antigen Mismatch (%0 Antigen Mismatch, N = 597)	35.6	39.9	38.4	0.75
B Antigen Mismatch (%0 Antigen Mismatch, N = 601)	29.5	36.5	33.9	0.12
DR Antigen Mismatch (%0 Antigen Mismatch, N = 597)	25.3	27.6	26.8	0.56
**Transplant procedure characteristics**				
Cold Ischemia Time (min; N = 551)*	1186 ± 745	1327 ± 746	1275	0.03
Delayed graft function (%; N = 615)	19.9	22.3	21.5	0.48
Induction protocol (%, N = 615)*	26.2	25.1	25.5	0.76

*p < 0.05 To convert Total bilirubin from mg/dL to μmol/L, multiply by 17.1; cholesterol from mg/dL to mmol/L, multiply by 0.02586; triglycerides from mg/dL to mmol/L, multiply by 0.01129; creatinine from mg/dL to μmol/L, multiply by 88.4.

### Predictors of statin use

In the multiple logistic regression, older age (OR 1.02; 95% CI, 1.00-1.04), elevated BMI (OR 1.08; 95% CI, 1.03-1.13) and higher cholesterol levels (per 10 mg/dL) (OR 1.08; 95% CI, 1.03-1.13) were associated with a higher likelihood of statin use, while higher alkaline phosphatase levels (OR = 0.995; 95% CI, 0.0.991-0.999) and CMV IgG positivity (OR 0.64; 95% CI, 0.39 - 1.05) associated with a lower likelihood of statin use. Statin use increased over the study period; in order to adjust for this, we included both the year of transplant and the sequential transplant identification key into the regression model, and the predictors for statin use did not change (Table 2). In our analysis of predictors of statin use, a number of patients lacked data for one or more variables. No substantial change in parameter estimates was seen when substituting average values for missing data. Steroid use was not associated with increased statin use. Sixty-three percent of the patients in this study were received maintenance steroids. The use of statins increased over time despite the decreasing use of steroids.

**Table 2:** Predictors of statin use among kidney transplant recipients

Variable	Crude Odds Ratio (95% CI)*	p-value	Adjusted Odds Ratio (95% CI)	p-value
Age at transplant (per year)	1.02 (1.007 - 1.032)	0.002	1.02 (1.00 - 1.04)	0.005
Body Mass Index	1.05 (1.02 - 1.09)	0.002	1.08 (1.03-1.13)	0.001
Sex	0.96 (0.68 - 1.35)	0.82	1.12 (0.71-1.76)	0.63
Race	1.51 (0.95-2.40)	0.09	1.23 (0.67-2.24)	0.50
MI	1.85 (1.00-3.42)	0.05	N/A	
Diabetes with complications	1.61 (1.05-2.47)	< 0.0001	N/A	
Alkaline phosphatase (per U/L)	0.99 (0.99 - 0.99)	0.02	0.99(0.99-0.99)	0.017
CMV IgG positive	0.63 (0.43 - 0.93)	0.02	0.64 (0.39 - 1.05)	0.08
Triglycerides (per 10 mg/dL)	1.004 (1.002 - 1.005)	< 0.0001	N/A	
Cholesterol	1.01 (1.005-1.014)	< 0.0001	1.01 (1.005-1.015)	< 0.001
Previous Transplant	0.68 (0.45-1.04)	0.07	N/A	

### Association of statin use with patient and graft survival

In order to avoid an immortality time bias, we restricted patient and graft survival analyses to the 615 grafts that survived at least one year from transplantation. During an average follow up of 2392 days beyond the first year after KTX, 152 of 615 kidney transplant patients died. During an average follow up of 1963 days, 253 of 615 kidney grafts failed (including failure because of death). As shown in Table 3, there was no significant association of statin use with patient survival in unadjusted analyses (HR 0.99; 95% CI: 0.72-1.37). The association remained non-significant after adjustment for age, race, BMI, BMI^2^, CMV IgG seropositivity, induction protocol, creatinine at twelve months, history of myocardial infarction, and severe liver disease (HR 0.86; 95% CI: 0.60-1.22). We also examined the association between statin use and graft survival after adjusting for serum creatinine at 12 months. Similarly, statin use was not associated with significant improvement in graft survival (HR 0.97; 95% CI 0.76-1.24). Even after adjustment for potential confounders such as baseline renal function as determined by creatinine at 12 months, history of myocardial infarction, severe liver disease, BMI and BMI^2^, statins failed to be associated with improved graft survival (HR 0.93; 95% CI 0.71-1.22). The propensity score analyses demonstrated slightly lower hazards associated with the use of statins and slightly narrower confidence intervals. However, the risk of death and graft loss associated with statin use in the full models was very similar to that demonstrated by the use of the propensity scores. Time to first acute cellular rejection (HR = 1.16; p = 0.21), after adjustment for recipient age, race, sex, BMI, PRA, donor type, donor age, antigen matching, and transplant procedure characteristics, was not associated significantly with the use of statins.

**Table 3:** Association of Statin use with Patient Survival and Graft Survival

	Patient Survival	Graft Survival
**Crude**	0.99 (0.72,1.37)	0.97 (0.76,1.24)
**Adjusted^1^**	0.86 (0.60,1.22)	0.93 (0.71,1.22)
**Weighted propensity^1^**	0.86 (0.68,1.09)	0.98 (0.82,1.18)
**Continuous propensity score^2^**	0.82 (0.58,1.25)	0.90(0.70,1.17)
**Quintiles of Propensity score^2^**	0.81 (0.57,1.15)	0.89 (0.68,1.16)

1 age, sex, race BMI, BMI2, donor type, previous transplant, CMV IgG positivity, tolerance protocol, creatinine at twelve months, myocardial infarction, severe liver disease

2. Previous transplant, creatinine at twelve months, myocardial infarction, severe liver disease

## Discussion

Data on early statin use in kidney transplant patients receiving tacrolimus are lacking, and this observational study adds valuable information to existing knowledge. In our large single-center cohort of kidney transplant recipients, 36% of patients used statins within the first year after transplantation, although statins were increasingly used over the course of our study period. The goal LDL target has also evolved over time. The mean LDL at 12 months among statin users in our study was greater than 2.59 mmol/L. We observed greater use of statins with older age, history of myocardial infarction, diabetes, higher BMI, and higher serum triglyceride and cholesterol concentrations. However, older age, elevated BMI and higher cholesterol levels remained independent predictors of increased statin use while CMV IgG positivity and higher alkaline phosphatase levels were predictors of lower statin use in the multivariate analysis. While the exposure to statins following kidney transplantation seemed appropriate, statin use within the first year following kidney transplant was not associated with better patient or graft survival. Even after adjustment for multiple variables including age, history of myocardial infarction, diabetes, sex, baseline creatinine and use of an induction protocol, statin use, as prescribed to our patients in this study, was not associated with improved patient or graft survival. In spite of the potential immunologic benefits of statin therapy on T-cell function, we did not find a difference in time to first rejection in patients receiving statins.

These findings expand existing knowledge by examining the effect of statins in a recent population of new kidney transplant patients largely managed with a tacrolimus-based immunosuppressive regimen. The broad inclusion of all kidney transplantation events at our institution, and the use of any statin within one year of transplant lend generalizability to our results. We were also able to evaluate the effect of baseline patient comorbidity on patient and graft outcomes in the setting of statin use by examining the Charlson comorbidity index, an instrument which has been previously validated in the kidney transplant population[29].

While only 36% of patients were using statins within the first 12 months after transplantation, it is also important to note that the majority of patients included in our study were treated prior to the published AST/ASTS clinical practice guidelines regarding the management of dyslipidemias in transplant patients[31]. In addition, underuse of statins has been reported in the general population[32,33]. The predominant use of tacrolimus at our institution may also partially explain the relatively low incidence of statin use in our patient population, as tacrolimus is known to be less likely to cause dyslipidemia compared to cyclosporine[26]. The relatively low prevalence of statin use is similar to that observed in a recently published observational study[6]. In addition, transplant patients are often on multiple medications, and because of concerns of drug-drug interactions and side effects, physicians may be less likely to prescribe statins for these patients.

In our study, statin use was not associated with improved graft survival, decreased incidence of acute rejection, or time to first rejection. These findings are consistent with results of the only randomized controlled trial examining statin use in kidney transplantation, the ALERT trial, as well as an observational study based on Austrian data. Our study confirms these findings in patients receiving primarily tacrolimus based immunosuppression. In addition, our study shows that early use of statins within one year after transplantation does not improve graft survival. Our findings differ from previous findings in non-transplant kidneys which have suggested that statins stabilize glomerular filtration rate (GFR) in patients with advanced kidney dysfunction[34]. We hypothesize that a potential small immunologic or non-immunologic benefit of statins on transplanted kidneys may be overwhelmed by other factors associated with poor renal function in those patients with suboptimal baseline graft function, and that the mechanisms underlying progression of kidney disease in this setting may differ in both cause and character from the course of kidney disease in native kidneys.

The number of studies examining the role of statins in kidney transplant patients as well as patients with advanced kidney disease is limited. The ALERT trial is the only randomized controlled trial of statin use in kidney transplant patients, and it failed to show an improvement in the primary outcome of major adverse cardiac event with the use of statins. This may have been due to inadequate study power as an open-label 2 year extension of the ALERT trial showed that patients randomized to the fluvastatin group had a reduced risk of major adverse cardiac events. Another possibility is that the pathophysiology of cardiovascular disease may be different in patients with advanced kidney disease compared to the general population. A randomized controlled trial of atorvastatin in patients with end stage renal disease on hemodialysis did not show improved survival in patients receiving atorvastatin(2). On the other hand, some observational studies have shown improved patient survival with the use of statins(5). Our study did not demonstrate improved patient survival with the use of statins. The reasons underlying the discrepant findings regarding the usefulness of statins in the transplant population remain poorly defined. Because of the limited number of available kidney transplant study subjects, studies examining the effect of statins in this population are often underpowered. Another possible reason for the lack of strong evidence favoring statin use in patients with advanced kidney disease is that cardiovascular disease may already be advanced, and the cardiovascular mortality risk effectively irreversible at the time of initiation of statin therapy. In addition, the pathophysiology of cardiovascular disease may be unique in the kidney transplant population, and non-traditional risk factors, which are not targets for statins, may play a larger role.

Our results must be interpreted in light of several limitations. We were unable to examine factors such as pre-transplant treatment with statins, reasons for initiating and discontinuing statin therapy. The full lipid profiles are not available for many of our patients. The potential for relatively short statin use is an additional limitation of our study; however, 89.9% of patients in the statin group exceeded the minimum criteria we defined for statin use. In addition, the indication for statin use was not uniform across all patients. Statins were not only prescribed by the transplant team but also by patients' personal physicians, including primary care physicians and specialists. While reflective of real-world practice, differing prescribing patterns may have affected the results of the study. Finally, the untreated group, who despite receiving no therapy, showed a trend toward lower lipid levels than the treated group. While the indications and goals of cholesterol management have evolved over the course of our study period, and the use of statins in our study may have been less aggressive than current practice, this finding raises the possibility that the two groups are systematically different and that our results are subject to confounding by indication.

Since these findings are from a single-center where patients are followed closely by the transplant group for the first six months and then at least yearly (but often much more frequently), thereafter, in conjunction with the referring nephrologists and primary care physicians, our findings may have limited application to other models of post-transplant care. The intensity of follow-up by the kidney transplant team is also impacted by graft function, and any complications related to transplantation. The outcomes of non-fatal MI, stroke, or cardiovascular hospitalization were not assessed. Finally, like other statin studies on kidney transplant patients, this study may also be underpowered to detect a small effect size of statins on patient and graft survival. However, our sample size calculations indicate that a sample size of 615 patients and a follow up period of 6.6 years provides relatively sufficient power to detect a major difference in patient or graft survival between the groups.

It is clear that there are differences among the various statins in terms of structure, derivatives, metabolism, potency, and influence on T cell function[10,11]. We were not able to draw any meaningful conclusions regarding differences between various statins from our patient sample. However, we did not find a decrease in the incidence or time to first rejection among patients receiving statins.

## Conclusion

While studies examining the effect of statins on the outcomes following renal transplantation vary in their conclusions regarding the presence and size of a putative beneficial effect, all studies to date support the position that this class of drugs can be used safely in the transplant population and has lipid-lowering benefits similar to those observed in other patient populations [35-39]. Of patients who die with a functioning graft, cardiovascular disease remains a major cause of death; the potential cardiovascular benefits derived from the use of statins, by themselves, prompt consideration of their use. The current AST-ASTS practice guidelines recommend lipid-lowering therapy for all kidney transplant recipients with fasting triglyceride levels ≥ 5.65 mmol/L, LDL cholesterol ≥ 2.59 mmol/L, or non-HDL-cholesterol ≥ 3.36 mmol/L, regardless of cardiovascular status, and notes that statins are the most effective class of antilipemic drugs for lowering LDL. However, we did not find a significant influence of statin use on patient survival; our findings suggest that the pathophysiology of cardiovascular disease in kidney transplant patients may differ from that of the general population and that these patients may have unique risk factors which are not targeted by statins. Therefore, there is still a need for large, well designed randomized trials in kidney transplant patients to establish a positive role of statins in this particular population.

## Competing interests

JJ has stock in Merck and Pfizer. Otherwise there are no competing interests.

## Authors' contributions

NY drafted manuscript and devised analysis plan, CW assisted with conception of the study design, drafted manuscript and devised analysis plan, RS drafted manuscript and participated in study design, JM drafted manuscript and participated in study design, JJ drafted manuscript and participated in study design, HT drafted manuscript and participated in study design, AB drafted manuscript and participated in study design, HS drafted manuscript and participated in study design, CS drafted manuscript and participated in study design, WW drafted manuscript and participated in study design, MU conceived of the study, drafted manuscript and performed the statistical analysis. All authors read and approved of the final manuscript.

## Pre-publication history

The pre-publication history for this paper can be accessed here:

http://www.biomedcentral.com/1471-2369/11/5/prepub
